# A Schutzian Analysis of Prayer with Perspectives from Linguistic Philosophy

**DOI:** 10.1007/s10746-015-9377-x

**Published:** 2016-04-06

**Authors:** K. Hoshikawa, M. Staudigl

**Affiliations:** 1 Department of Humanities, Taisho University, Tokyo, Japan; 2 Department of Philosophy, Vienna University, Vienna, Austria

**Keywords:** Schutz, Wittgenstein, Prayer, Christianity, Speech act, Language-game, Multiple realities

## Abstract

In this paper, we propose to analyze the phenomenon of Christian prayer by way of combining two different analytical frameworks. We start by applying Schutz’s theories of “intersubjectivity,” “inner time,” “politheticality,” and “multiple realities,” and then proceed by drawing on the ideas and insights of linguistic philosophers, notably, Wittgenstein’s “language-game,” Austin’s “speech act,” and Evans’s “logic of self-involvement”. In conjoining these accounts, we wish to demonstrate how their combination sheds new light on understanding the phenomenon of prayer. Prayer is a complex phenomenon that involves two major dimensions: the private and the social, as Matthew (6: 6) and Acts (1: 14), respectively, demonstrate. Schutz’s study of the phenomenon of “inner time” and the “polithetical” structure of consciousness, at both the subjective and intersubjective level, provides a useful lens to analyze these two dimensions. In addition, prayer, in following a specific set of rules, can also be considered as a specific, i.e., religious “language-game”. In the last analysis, however, we propose to analyze prayer (and, finally, religion) within the Schutzian framework of “multiple realities,” “enclaves,” and “symbolic appresentation,” which permits accessing the “religious finite province of meaning” in the very midst of the paramount reality of everyday life. In a nutshell, we claim that Christian prayer is a practice of constructing and living within a “religious province of meaning” in the everyday world; it is a practice that revolves around self-involving language-activities such as praising, confessing, thanksgiving, or requesting to God, which enable the praying subject to transfigure the language of everydayness and “see through” (Schutz) the world of everyday life in order to let it appear in a different light, e.g., the light of grace, gift, and salvation.

## Introduction

Whereas phenomenology bears an analytical focus on “consciousness” as its central theme, linguistic philosophy analyzes “language” as its central theme. Without a doubt, these two traditions have not been harmonious or sometimes have even been opposed to each other for nearly 100 years. According to our understanding, however, they can be consolidated very well when being applied to concrete analyses: in this essay, we shall demonstrate this for the analysis of “prayer” which indeed consists of *two aspects*, namely, a *conscious* aspect and a *linguistic* one.

Firstly, prayer emerges in the world of daily life and constructs its meaningful “field” or “enclave” in the very midst of this world. In other words, prayer jumps out of the horizon of the flow of consciousness into the core of an experience of “transcending everydayness”. As to our hypothesis, we can analyze this process very well from the perspective of Schutzian phenomenology. Secondly, prayer—being realized in acts of praising, confessing, thanksgiving, or requesting to God—is definitely a linguistic activity, i.e., a “performative” or “self-involving” activity. In a second step, we will analyze this aspect from the perspectives of Austin and Evans. Finally, however, we propose to come back to Schutz: his account of “finite provinces of meaning” offers viable conceptual devices to give an account of the capacity of prayer to “see through” the world of everydayness, to transcend its cognitive style, and to partake in a logic (e.g., the logic of grace, salvation, redemption, etc.) that escapes the limitations of the “pragmatic motive”. Since, as Schutz emphasizes, “it is the meaning of our experiences, and not the ontological structure of the objects, which constitutes reality” (Schutz 1945b: 341), this implies that the meaning of experiences in prayer constitutes its reality. This meaning undoubtedly is connected with language but also with its constitution in consciousness, which, according to Schutz, has to be understood in terms of an inherently embodied intentionality. *The reality of prayer*, *thus*, *is co*-*constituted by human consciousness and language*, a fact which shall be demonstrated by recourse to Schutz’s theory of “finite provinces of meaning”.

Viewed against this background, we propose to combine these two qualitatively different philosophical disciplines in order to analyze the religious phenomenon of prayer, which, according to various commentators, has to be located at the core of religion. As a by-product, we wish to demonstrate that the two traditions need no longer be considered to be opposed to each other, but can very well be incorporated by way of concrete analyses—prayer being the issue at hand.

## Contemporary Religion and Prayer as the Core/Essence of Religion

A historical review of the world’s religions reveals the practice of various forms of prayer. In this article, we focus on those of Christianity in the context of the present, secularized age in which Berger’s “Sacred Canopy” is of fading significance.

The secularization of religion has been a topic of great debate among sociologists of religion since the 1960s. Though religious consciousness has been intensifying in some areas, and among some people, secularization has been promoted in many of the world’s big cities and urban neighborhoods as a result of prevailing ways of thinking and knowing, including those of the natural sciences.

Since the 1990s, because of the development of medical machines such as PET, fMRI, SPECT or MEG, the neurosciences have made great progress. Neuroscientists have begun to delve into describing religious experiences during meditation, prayer, or “encounters” with God. While some outstanding neuroscientists (for example, Benjamin Libet and Nobel laureate John Eccles) were devoutly religious (Jewish and Christian respectively), many other neuroscientists, including another Nobel laureate, Francis Crick, either do not profess religious faith or are agnostic. Many materialistic neuroscientists reduce religious experiences to the physical functions of the brain. Their conclusion appears to be that those who are religious can have religious experiences even if God does not exist.

Apart from neuroscientists, Cupitt, a philosopher of religion, has pointed out the necessity of a radical change or paradigm shift in religious thinking:

If all our received religious traditions have now come to an end and a Second Axial Age [sc., an epoch in which we must be prepared to rethink everything] has perhaps already begun, then we need radically new religious thinking. We need, for a time at least, to abandon the old institutions, the old doctrines, the old vocabularies, and even the old assumptions about what religion is. Thus ‘pure’ religious thinking is a post-traditional kind of thinking that breaks with the past and attempts to make a new beginning. (Cupitt 2001: 9)

[…] we have to forget all forms of the notions that something supremely Real out there [sc., God] controls reality and grounds all of our knowledge and values. On the contrary, what we see as being out there [sc., God or supreme Reality] is only our own ever changing projection [sc., of language]. There is no objective reality, no objective truth, no objective value, and no objective or absolute knowledge. (Cupitt 2001: 14)

Furthermore, Cupitt stresses the supreme role of language, given that everything is constructed through our use of language:

We don’t need metaphysics. Look around you: what you see is only sense experience which has been formed as such by our language, and by language (including the language of our theory) has been stitched together to make a continuous world. *We* assemble it all [sc., by language], and *we* give to it its apparent ‘reality’ [sc., by language]. (Cupitt 2001: 9f.)

We cannot know objectively whether God as reality itself exists or not. But Cupitt’s viewpoint reflects one of the notable trends of modern thought. In any case, we could say that even if God does not exist as objective reality, people can still talk about and believe in God as “an intentional object” that is constructed by language.

Whereas in the present era we are not mandated to believe that a supreme God controls everything, Schutzian perspectives are still useful for analyzing the phenomenon of prayer. The reasons are as follows. First, even if we cannot know whether God really exists, we can construct “religious reality” through language. Second, although the neurosciences have advanced greatly, research in this field has not yielded any essential insights on “consciousness,” with which Schutz is deeply concerned. Thus, consciousness remains—despite all attempts at (re)naturalizing it—shrouded in mystery. Third, the Schutzian and neuroscientific approaches to prayer differ, or entail different and autonomous “language-games”. A Schutzian approach to consciousness cannot be reduced to a neuroscientific one.

Heiler, the author of the classic *Prayer*: *A Study in the History and Psychology of Religion* (1932), considers prayer to be the central feature of religion. Taking an expansive view of religious adherents, including religious scholars and theologians of all creeds and tendencies, he states that all of them concur that “prayer is the central phenomenon of religion, the very hearthstone of all piety”. Faith is, in Luther’s judgment, “prayer” and “nothing but prayer”. We should, moreover, note that even Feuerbach, the most radical of the critics of religion, declares that “the innermost essence of religion is revealed, by the simplest act of religion—prayer” (see Heiler 1932: xiii–xv).

Heiler quotes a number of views on prayer put forward by well-known individuals versed in Christian traditions. Those cited here are unanimous in their confirmation of prayer as a central feature of religion. Arndt, a great Protestant mystic, repeatedly emphasizes that “without prayer we cannot find God; prayer is the means by which we seek and find Him”. Schleiermacher, who revived Protestant theology, observes: “To be religious and to pray—that is really one and the same thing”. Rothe, also a Protestant theologian, states: “the religious impulse is essentially the impulse to pray. It is by prayer, in fact, that the process of the individual religious life is governed, the process of the gradual fulfillment of God’s indwelling in the individual and his religious life […]. Therefore, the non-praying man is rightly considered to be religiously dead”. Deissmann, another Protestant theologian, holds that: “religion, wherever it is alive in man, is prayer”. Tiele, one of the founders of the science of comparative religion, opines: “Where prayer has wholly ceased, it is all over with religion itself”. Sabatier, a philosopher and religious scholar, says: “Where the prayer of the heart is wanting, there is no religion”. Lastly, Hettinger, a Catholic apologist, describes prayer as “the first, highest, and most solemn phenomenon and manifestation of religion” (see Heiler 1932: xiii–xvi).

Based on these views, Heiler concludes as follows:

Accordingly, there can be no doubt at all that prayer is the heart and centre of all religion. Not in dogmas and institutions, not in rites and ethical ideals, but in prayer do we grasp the peculiar quality of the religious life. In the words of a prayer we can penetrate into the deepest and the most intimate movements of the religious soul. (Heiler 1932: xv)

More recently, Chrétien, the author of *The Ark of Speech* (2004), starts a phenomenological study of prayer with the following words:

Prayer is the religious phenomenon par excellence, as it is the human act that alone opens up the religious dimension and never ceases to sustain, to bear, to suffer that opening […]. With prayer the religious phenomenon begins and ends. (Chrétien 2004: 17)

## Phenomenology and Prayer

In what follows, we will focus on two types of prayer: solitary prayer and collective prayer with others. An example of the former is mentioned in Matthew (6: 6): “But whenever you pray, go into your room and shut the door and pray to your Father who is in secret”.^1^An example of the latter occurs in Acts (1: 14): “All these [people] were constantly devoting themselves to prayer, together with certain women, including Mary the mother of Jesus, as well as his brothers”. These two types of prayers are closely connected with and supportive of each other.^2^ We suggest that Schutzian perspectives are helpful for investigating those two aspects of prayer, since they allow for reflecting both the subjective and intersubjective aspects of consciousness.

Schutz’s analysis of the “interplay” between subjective and intersubjective experience requires careful examination. His discussion in “Making Music Together” (1951), which aims to provide a foundation for transpersonal experience, is concrete and persuasive. He refers to Wiese’s “contact-situations,” Scheler’s perceptual theory of the alter ego, and Sartre’s core concept of “looking at the Other and being looked at by the Other,” etc. Schutz relates all these accounts to his endeavor to investigate the “mutual tuning-in relationship” on which alone all varieties of communication are founded. In Schutz’s words: “It is precisely this mutual tuning-in relationship by which the ‘I’ and the ‘Thou’ are experienced by both participants as a ‘We’ in vivid presence” (Schutz 1951: 161).

Prayer, fostered in a religious community or society, consists of words, phrases, and sentences that are conceptually constructed by this group. Viewing prayer as a collection of concepts emphasizes its social construction. However, focusing specifically on religious experience (not religious images, symbolic systems, or cosmologies), Spickard (1991: 193) criticizes Berger’s and Luckmann’s interpretation of Schutz, because they read him through a Sumnerian or Durkheimian lens that precludes consideration of religious experience *per se*. Religious experience is a subjective phenomenon, therefore we must not cut off the experience from the realm of experiencing subjectivity. Spickard summarizes Husserl’s phenomenology and Schutz’s application of it to sociology as follows:

Husserlian phenomenology attempts to ground secure knowledge in […] subjectivity by means of a rigorous examination of experience. In particular, Schutz’s application of phenomenology to sociology is an attempt to highlight the interplay between social life and experience—not to demonstrate the hegemony of the former. Schutz certainly thought of himself as a phenomenologist of the Husserlian school. (Spickard 1991: 193)

The “mutual tuning-in relationship,” that is, the very possibility of shared experience, is closely related to the “polythetic” constitution of our experience. Schutz provides the following description of such a polythetic interrelation of recollections, retentions, protentions, and anticipations by reference to a piece of music:

For our purposes a piece of music may be defined […] as a meaningful arrangement of tones in inner time […]. The flux of tones unrolling in inner time is an arrangement meaningful to both the composer and the beholder, because and in so far as it evokes in the stream of consciousness participating in it an interplay of recollections, retentions, protentions and anticipations which interrelate the successive elements. (Schutz 1951: 170)

Adopting the same perspective, Spickard, emphasizing the polythetic aspect of prayer, notes:

Like music or poetry, prayer is a polythetic phenomenon. It presents a stream of images that structure inner time. It guides the hearer from image to image: backward as the images repeat what has been, forward as they foretell what is to come. Where theology is meant to convince, ritual prayer is meant to be experienced. (Spickard 1991:200)

People who devote themselves to prayer are apparently experiencing—or rather: living through—such a polythetic process. This can be illustrated through the “Lord’s Prayer,” which is a central prayer in Christianity appearing in the two Gospels of Matthew and Luke. In the Gospel of Matthew (6: 9–13) Jesus states:

Pray then in this way: Our Father in heaven, hallowed be your name.

Your kingdom come. Your will be done, on earth as it is in heaven.

Give us this day our daily bread.

And forgive us our debts, as we also have forgiven our debtors.

And do not bring us to the time of trial, but rescue us from the evil one.

When Christians pray: “Give us this day our daily bread,” some of them might be recollecting a past time when food was scarce, or anticipating a future time when they will receive daily bread. When they pray, “forgive us our debts,” they might be reflecting on their intended acts and deciding not to commit that crime again. When they pray, “rescue us from the evil one,” they might be remembering an unhappy past and imagining a peaceful future. During prayer, as Schutz mentions, believers experience the interplay of recollections, retentions, protentions, and anticipations, whereby successive elements are interrelated on a largely pre-reflective level. Or, as Spickard suggests, prayer may be seen to provide guidance from image to image: backward as the images repeat what has been; forward as they foretell what is to come.

The world of daily life pre-exists our birth and is experienced and interpreted by us as an organized, i.e., socially derived and pre-structured world. In short, this meaningful constitution of the social world is the basis of all our experiences, thinking, and actions. Schutz developed an integrated phenomenological approach for examining the “world of daily life,” which is also commonly referred to as “the world of everyday life,” “the everyday world,” “the paramount reality,” “the paramount world of real objects and events,” or “the Life-world,” if taken from a mundane standpoint. In this context, he enumerated six characteristics of this world as follows: (1) A specific tension of consciousness, namely, “wide-awakeness,” which originates in an attitude of full attention to life. (2) A specific *epoché*, namely, the suspension of doubt. (3) A prevalent form of spontaneity through working or gearing-into the world (*Wirken*). (4) A specific form of experiencing one’s self (the working self as the total self). (5) A specific form of sociality (the common intersubjective world of communication and social action). (6) A specific time-perspective (standard time originating in an intersection between *durée* and cosmic time as the universal temporal structure of the intersubjective world) (see Schutz 1945a: 230f).

Viewed against this background, the “field” wherein prayer is practiced can be understood as an “enclave,” which is unfolded by human consciousness in the midst of the world of daily life. By enclave, Schutz points at the overlapping of various realities or the fact that “regions belonging to one province [are] enclosed by another” (Schutz 1945a: 233 note); they are, as he also puts this, looped into “foreign territory” (Schutz 1945b: 307). Thus, we may describe an “enclave” as a privileged site or as an institutionalized point of entry into another “finite province of meaning,” which introduces or prescribes the “particular cognitive style” and “attentional attitude” (Schutz 1945a: 233 note) necessary in order to enter another reality.^3^

Schutz explains that each finite province has “its particular style of a set of our experiences”. This includes: (1) a specific tension of consciousness, (2) a specific time-perspective, (3) a specific form of experiencing oneself, and (4) a specific form of sociality. The enactment of such a particular cognitive style or attitude (e.g., by variously bracketing the relevancies of everydayness and entering the worlds of theory, art, religion, etc.) constitutes the enclave (e.g., the laboratory, the theater, the temple, etc.) as the privileged and habitual site of accessing another finite province of meaning in which all experiences appear consistent in themselves and compatible with one another (see Schutz 1945b: 341). Yet, this finiteness implies that “there is no possibility of referring one of these provinces to the other by introducing a formula of transformation” (Schutz 1945a: 232). The passing from one province to another can only be performed through what Kierkegaard refers to as a “leap,” which “manifests itself in the subjective experience of a shock” (Schutz 1945a: 232).

To return to our example: Christians pray to God because they believe that He ordered them to do so and promised to listen to their voices. Thus, prayer came into existence as a response to this call from God. The field, reality, or enclave of prayer can consequently be understood as one whose nature is completely different from that of the world of daily life. Referring to Schutz’s earlier cited statement, there is no “formula of transformation” between these worlds.

The exposition of Augustine Ichiro Okumura, a Japanese Carmelite, resonates well with Schutz’s explanation of the relation between the world of daily life and the enclave or finite province of prayer. He interprets prayer as “breaking off all relations with daily affairs”^4^ (Okumura 1974: 126) and states:

What we have to study in prayer is firstly to die, to realize the emptiness of ourselves. Facing with God who is infinite and eternal, we have to see the very transitory nature of ourselves as it is and study to die in/with God. These things should lie at the bottom of prayer. We must have the experience of death even during short-time prayer. While we are devoting ourselves to prayer, we must never think of ordinary life. (Okumura 1974: 123)

In this context, “to die” means to break off or inhibit all relations with the world of daily life. The analogy of this procedure with the phenomenological epoché is apparent; accessing the religious province of meaning thus entails a sort of “religious epoché”.

Furthermore, Okumura states: “While we are engaging in prayer, we can be said to transcend time within the stream of standard or objective time, and transcend the space within it. In other words, we transcend the world, while staying in it” (Okumura 1974: 135). For him, the most important principle of prayer is “to cut off the stream of consciousness of daily life at the right angles, as a bamboo marks off itself with its joints” (Okumura 1974: 125f.). We should not bring daily affairs into prayer. “To die,” in the sense used by him, conveys all these meanings and actions. Breaking off all relations with the daily world is the “breaking bravely with daily life whose vacancy we know” (Okumura 1974: 145).

It may not be possible for a non-Christian to understand what Okumura is trying to convey, because it sounds contradictory. As we know, however, religious teachings quite often transcend the usual modes of thinking and understanding. Referring once more to the earlier statement by Schutz, there is no reference through a “formula of transformation” between the religious province of meaning opened up by prayer and that of daily life.

In other words, the departure from the world of daily life can be understood as one of coming out of that world and entering into the religious province of meaning via the enclave of prayer. Given that no “formula of transformation” exists between these two worlds, when a Christian breaks off relations with the world of everyday life to pray, he or she must do so, as Okumura emphasizes, in the same way as a bamboo marks off itself with joints at right angles. This implies that a person transits from the province of the everyday world to that of prayer by shifting the accent of reality, or by changing the direction of the stream of consciousness. This “transition,” however, must not be misinterpreted as a sort of real migration from one ontological level to another, but as a modification or alteration of the perception and evaluation of the everyday world.

Prayers are sometimes said by individually motivated believers, sometimes they are collectively brought forward by a group. In both cases, believers respond to the vacancies, inconsistencies, or deficiencies of everydayness by addressing a new theme or reality in prayer; in transforming given meaning-structures they propose to “see through” the relative naturalness of the paramount reality one lives in without reflecting upon it. In other words, the world of prayer intrudes into the horizon of the believer’s flow of experience, breaching its quasi-ontological homogeneity. In this process, the lived unity of our ways of perceiving the everyday world collapses and the new vision endorsed in prayer becomes thematic and may, finally, receive the accent of reality. Prayer, put differently, “jumps out” of the horizon of the flow of experience into the very core of experience, thus opening pathways to transfigure our predominant ways of experiencing. At the same time, the dominant theme or relevance that captures one’s attention in the everyday world—e.g., working hard, driving a car, or chatting with friends—which has prevailed unanimously until this time, is abandoned (see Schutz and Luckmann 1974: 186–190) and something “wholly other” comes to mind.^5^

The processes carried out before, in, and after prayer can be described as follows. In the first place, Christian believers who have not yet committed to prayer are living in the everyday world. Having suspended any doubt of that world, they take it for granted, living their usual social lives that rely on common sense, communication with others, social roles, and the standard time of the intersubjective world—all this taking place under the yoke of the “pragmatic motive” that structures our average existence. Those who are about to devote themselves to prayer—for whatever reasons—begin to shift their interest and focus from the world of daily life and social business as usual to one of prayer. In this situation, religious consciousness or tension gradually mounts. As this process continues, the activity of prayer, figuring as a portal to the religious province of meaning, becomes predominant; the person praying becomes immersed in the religious “province of meaning,” which entertains no ontological relations with the world of daily life but rather sheds a new light on its very givenness and the way we are used deal with it. Given this, the everyday world of collectively shared, secular common sense and standard time fades away and the religious “reality” becomes the primary site of rendering the everyday world intelligible otherwise, e.g., in the light of grace, the gift of love or salvation, or the promise of unconditional hospitality, etc.

Occasionally in the world of prayer, eternal communion, dialogue, and personal contact with God might come to be realized, but a full-fledged description of such “achievements” of prayer is not the focus of this paper. Towards the end of the invocation, religious consciousness, mindfulness, attentiveness or tension begins to fade out, and the person praying returns to the everyday world. This process, however, repeats and sediments itself in the stream of his/her consciousness at an unpredictable frequency that not only depends on the believer’s outlook, but transforms it, too.^6^

## Prayer and Linguistic Philosophy

From the preceding discussion, we can conclude that the reality of prayer, viewed as an enclave that opens access to the religious finite province of meaning, is constituted by a specific attitude of practicing consciousness: the significance of prayer has been located in its capacity to change the direction of the stream of consciousness from the world of daily life to the religious finite province of meaning. In a next step, we will now consider the relations that exist between reality and language in this context.

We should note that Schutz distinguishes his “finite province of meaning” from James’s “sub-universe” in *Principles of Psychology* (1890), emphasizing that “it is the meaning of our experiences, and not the ontological structure of the objects, which constitutes reality” (Schutz 1945b: 341). If the meaning of our experiences constitutes reality, then *the meaning of experiences during prayer constitutes its reality*, and *the meaning of experiences is*, *without a doubt, connected with language*. In short, *the language of prayer is essential for constructing its reality*. When the “language-game” of prayer is applied, the reality of prayer is constructed. Cupitt states that “there is no objective reality, no objective truth, no objective value, and no objective or absolute knowledge” validates this conclusion. Reality is constructed by human language, and even the reality of God could be interpreted as a projection of language.

Lindbeck, a Protestant theologian of doctrine, describes the construction of reality by language as follows:

[…] it [sc., a religion] is similar to an idiom that makes possible the description of realities, the formulation of beliefs, and the experiencing of inner attitudes, feelings, and sentiments […]. It comprises a vocabulary of discursive and nondiscursive symbols together with a distinctive logic or grammar in terms of which this vocabulary can be meaningfully deployed. (Lindbeck 1984: 33)

Thus, we can infer that the senses of reality and language are interdependent or, perhaps, one and the same.

With regard to our context, Schmidt notes that “For several 1000 years, people have thought that some religious assertions were factual claims. If our analysis is correct, however, this long tradition, still strong in some of our contemporary theologians, is mistaken […]. Religious claims do not belong to factual knowledge” (Schmidt 1968: 224). Wilson, in his analysis of religious language, classifies sentences employed in Christian literature, creeds, or rituals into five categories, namely: (1) sentences expressing commands, injunctions, exhortations, and wishes; (2) sentences expressing moral views; (3) sentences expressing factual, and often historical, truths; (4) sentences conveying information about the meanings of words, and expressing analytic truths; and (5) sentences that appear to be informative about the supernatural or metaphysical world, as opposed to the natural or physical world (Wilson 1968: 356).

As both Schmidt and Wilson [categories (1) (2) (5)] suggest, sentences used in the context of Christian literature, creeds, or rituals contain many elements which, quite often, have *nothing* to do with facts, logic, and verification. This observation equally applies in the case of prayer, which is at the core or essence of Christianity. The words, phrases, and sentences employed in prayer are in sharp contrast to those used in the natural sciences, factual reports, or rational reasoning. Regarding words used in prayer, the concept of “performative utterance,” which draws attention to the performative function of words or language, provides a helpful perspective for examining prayer.

Austin laid the foundation of “speech act” theory in his analysis of performative utterances expressed by verbs conjugated in the first person, singular, present, indicative, active. By the time when his philosophy came to be known, philosophers had long assumed that the purpose of a statement could only be to describe some state of affairs, or to state some fact, either correctly or falsely. He, however, argued that this was not the case. He analyzed performative utterances/sentences by attending closely to the functions of verbs such as “promise” or “name”. He contended that “the issuing of the utterance is the performing of an action—it is not normally thought of as just saying something”. The utterances “do not ‘describe’ or ‘report’ or constate anything at all, are not ‘true or false,’” and “the uttering of the sentence is, or is a part of, the doing of an action” (see Austin 1975: 1–7).

Drawing on Austin’s insights, Evans (1963) introduced the idea of performative utterances to the study of the philosophy of religion, viewing the language of Christianity as an expression of “self-involvement” and total commitment to God. He investigated various articulations of Christian language, which have explicitly or tacitly adopted the formula: “I acknowledge X as Y,” and interpreted religious utterances as “self-involving” when they were “behabitive” or “commissive” (see below). In analyzing these forms of language, he shed new light on the logic of the activity of commitment:

Older logics deal with propositions (statements, assertions); that is they deal with relations between propositions and relations between terms of propositions. Modern biblical theology, however, emphasizes *non*-*propositional* language, both in its account of divine revelation (God’s ‘word’ to man) and in its account of human religious language (man’s word to God). In each case the language or ‘word’ is not (or is not merely) propositional; it is *self*-*involving activity*, divine or human […] he [sc., man] addresses God in the activity of worship, committing himself to God and expressing his attitude to God. (Evans 1963: 14)

These concepts, namely Austin’s performative utterance and Evans’s self-involving utterance, are helpful for examining the language used during prayer. “Behabitives,” a kind of performative “concerned roughly with reactions to behavior and with behavior towards others and designed to exhibit attitudes and feelings” (Austin 1975: 83), are particularly fruitful for investigating Christian prayer. Their application supports a view of prayer in terms of its capacity to construct a religious reality.

To return to the “Lord’s Prayer,” let us recall that this prayer appears in two forms in the New Testament. The first is the previously cited version in the Gospel of Matthew (6: 9–13).The second version in the Gospel of Luke (11: 2–4) is as follows:

He [Jesus] said to them, “When you pray, say: Father, hallowed be your name. Your kingdom come.

Give us each day our daily bread.

And forgive us our sins, for we ourselves forgive everyone indebted to us. And do not bring us to the time of trial”.

The words and sentences employed in these two versions of the prayer are expressions of entreaty, desire,^7^ or aspiration toward God, which are reflected in the Lord’s Prayer as a whole. They are not factual statements as in the natural sciences or in our daily life, but rather can be viewed as examples of Austin’s “performative utterance” and Evans’s “self-involving utterance” in the broad sense of these terms.

Prayers involve a combination of various important elements, which are performative or self-involving, for example, “praising,” “confessing,” “thanksgiving,” “requesting,” etc. Some examples extracted from the Bible include: (1) Praise: “this is my God, and I will praise him, my father’s God, and I will exalt him” (Exodus 15: 2). (2) Confession: ^“^If we confess our sins, he [sc. God] who is faithful and just will forgive us our sins and cleanse us from all unrighteousness” (1 John 1: 9). (3) Thanksgiving: “Give thanks to the Lord, for he is good; his love endures forever” (1 Chronicles 16: 34). (4) Requesting: “Ask, and it will be given to you” (Matthew 7: 7).

To apply Austin’s insights to prayer, the words and sentences employed in praising, confessing, thanksgiving, or requesting do not in any way describe, report, or constate facts, and the uttering of these words and sentences is, or is a part of, performing an action. These are expressions of the believer’s attitude or commitment to God, and are self-involving performative utterances.^8^

A belief in God lies at the root of praying. Suppose, for example, that a Christian says, “I *believe* that God exists”. This statement is not a simple assertion that God exists, but rather an expression of the person’s total commitment to God. Saying that “I believe that God exists” is a kind of performative action with self-involvement. This implies that the believer pledges loyalty to God, following His moral legislation (concerning, for instance, the correct ways how to organize one’s community in the “imitatio Christi,” epitomized, e.g., in the figure of the “mystical body”). And the explicit issuing of the performative formula, “I acknowledge X as Y” implies that the subject has a positive attitude or intention toward X. For example, by stating that: “I *acknowledge* the Holy Spirit as the source of life,” the believer performs an action of acknowledgement and expresses an attitude of acceptance of the Holy Spirit as the source of life and a commitment to the Spirit (see Evans 1963: 150f.).

Furthermore, even if an explicit performative or self-involving verb such as “believe” or “acknowledge” is absent in sentences of prayer, these characteristics can be indicated. For example, when a person who is praying says, “God is my Creator,” while this sentence appears to be a factual statement, it is actually an abbreviated form of the sentence: “I *believe* that God is my Creator,” or “I *acknowledge* God as my Creator”. In short, the formula of “I believe (in) X” or “I acknowledge X as Y” permeates the language of Christian tradition. Furthermore, to say “I committed X” is not merely to state a fact, but also to *confess* the crime X and to *ask* God for forgiveness of X. To say, “my son is seriously ill” is not simply to state a fact, but also to *appeal* to or *ask* God to heal him. The language used in Christian prayer, hence, is of a highly performative or self-involving character.^9^

As to our understanding, the preceding ideas can be taken up with reference to Wittgenstein, and especially with regard to his concept of the “language-game”. In other words, we have thus far analyzed words of performative or self-involving character on the individual level of the individual word but will now proceed, in the following discussion, to examine the language of Christianity in terms of a “system” or “totality”. Wittgenstein, who was himself devoutly religious, has had a remarkable influence on philosophy of religion, especially within the analytic tradition. He wrote the following statement on June 11, 1916 when he was 27 years old and serving in World War I: “The meaning of life, i.e., the meaning of the world, we can call God. / And connect with this the comparison of God to a father. / To *pray* 10 [emphasis added] is to think about the meaning of life” (Wittgenstein 1961: 73). He later included “praying” as a final example of language-games in section 23 of his *Philosophical Investigations* (Wittgenstein 1958).

Furthermore, in *Philosophical Grammar* (Wittgenstein 1974), he stated: “When we study language we *envisage* it as a game with fixed rules. We compare it with, and measure it against, a game of that kind” (Wittgenstein 1974: sec. 36). This last statement by Wittgenstein can also be applied to Christianity, viewed as a system of language-games that is played with its own rules.

Wittgenstein’s insights enable us to expand on our analytic approach of Christian prayer, viewed as personal speaking, dialogue, or communion with God that follows a specific set of Christian rules.^11^ Prayer can be conceived as a language-activity or language-game within a finite province of meaning, which is brought about by our consciousness, and constructs a reality different from that of the world of daily life.

To validate this interpretation of Christianity as “a system of language-games,” excerpts from some sections of Wittgenstein’s *On Certainty* (1969) will be helpful. The various activities using language in Christianity are not disconnected but form a vast system of interconnected language activities. In his discussion on testing, confirmation, argument, and knowledge, Wittgenstein emphasizes the importance of a *system* for the conduct of these activities using phrases like “within a system,” “a totality of judgments,” or “a whole system of propositions”. His statements are, as we shall see below, compatible with Schutz’s theory of “multiple realities,” notably, that: “there is no possibility of referring one of these provinces to the other by introducing a formula of transformation”.

Wittgenstein states:

All testing, all confirmation and disconfirmation of a hypothesis takes place already within a system. And this system […] belongs to the essence of what we call an argument. The system is not so much the point of departure, as the element in which arguments have their life. (Wittgenstein 1969: sec. 105)

*A totality* of judgments is made plausible to us. When we first begin to *believe* anything, what we believe is not a single proposition, it is a whole system of propositions. (Wittgenstein 1969: sec. 140–141)

Our knowledge forms an enormous system. And only within this system has a particular bit the value we give it. (Wittgenstein 1969: sec. 410)

These statements can be applied to religion in the following way: (1) Christians believe in a whole system of religious propositions. (2) Within this system, each word, phrase, or sentence can have a meaning. (3) Religious knowledge forms an enormous system, and it is only within this system that a particular component (word, phrase, or sentence) has an assigned value.

Lindbeck further stresses the incommensurability of different language-games, even among the different religions or philosophies:

[…] the cultural-linguistic [sc. Lindbeck’s] approach is open to the possibility that different religions and/or philosophies may have incommensurable notions of truth, of experience, and of categorical adequacy, and therefore also of what it would mean for something to be most important (i.e., “God”). (Lindbeck 1984: 49)

Taking Wittgenstein’s emphasis on a system, totality, or wholeness into consideration, we may presume that what is real/unreal, true/false, or meaningful/non meaningful is determined within the whole system of language-games.^12^ And we can apply this interpretation to Christianity if we conceptualize it as a system composed of sub-systems of language-games, one of which is prayer (see below).

Many Christian philosophers or theologians, the so-called “Wittgensteinian Fideists,” who accept Wittgenstein’s above-mentioned insights, maintain that Christianity, as a whole, is a system of religious language-games based on the idea of God and a commitment to Him. Their intent is to protect Christianity from attacks by atheism, the natural sciences, or secular common sense, which do not admit the existence of God. Phillips, e.g., states: “the criteria of the meaningfulness of religious concepts are to be found within religion itself,” and “whether a mistake or confusion has occurred, is to be recognized by criteria found within religion” (Phillips 1965: 12). Evans, parodying the slogan of the ordinary language school, stresses that “the basic ‘ordinary’ language to which an analytic philosopher should appeal when he considers *Christian* conceptions is biblical language” (Evans 1963: 17).

All these Wittgensteinian points can indeed be applied to Christianity, viewed as a system of language-games that has its own logic or grammar. Different language-games have their own criteria regarding reality, truth, and meaningfulness. In other words, Christianity constructs its own system of language-games, which is qualitatively different from those of atheism, the natural sciences, and secular common sense. It constructs its own world and is ordered as it is.

A question that arises from this discussion is “how does Christianity, conceived as a system of language-games, relate to its central component of prayer?” We can understand *prayer* in terms of *a sub*-*system of the super*-*system of Christianity*. Words, phrases, or sentences employed in a prayer have appropriate meanings only in the context of Christianity. Inserting prayer into Wittgenstein’s previously cited statement, we may say that the practice of prayer takes place within a Christian system; that the system is not so much the point of departure as the *element* in which prayer has its life; and that only within the system does prayer have the value we give it.

The stated aim of this paper is to focus on Christian prayer in the present secularized age because, as Cupitt strikingly demonstrates, it seems almost impossible in the current era to provide Christian faith with a solid foundation. However, the answer to the question of whether or not Christian faith must have a solid ground or foundation to be believed or committed to by believers is “*no*”. Even if believers have no ground or foundation for their faith, they do not have to stop devoting themselves to Christianity.

Christianity, or its prayer, consists of framework propositions to which believers commit themselves, such as “God exists,” “God created this world,” or “God loves us”. Can Christians give us a rational reason or justification for these religious beliefs? As for the existence of God, someone might say that God has showed Himself within him/her, or that, as Paul says, “it is no longer I who live, but it is Christ who lives in me” (Galatians 2: 20). It is, however, very difficult in the present secular age to rationalize or justify belief in God. However, from the perspective of language-games, Christians, even if they cannot provide a rationale for the ground or foundation of their belief, can still believe in Christianity. In other words, the ground or foundation is neither necessary nor essential to play the language-game of Christianity, since it is “groundless”.

The process of providing foundations sooner or later comes to an end. Wittgenstein talks about this process of justification using the metaphor of a spade and bedrock: “If I have exhausted the justifications I have reached bedrock, and my spade is turned. Then I am inclined to say: ‘This is simply what I do’” (Wittgenstein 1958: 217). Thus, the process of justification, or providing a foundation, ends here. A believer who has reached this endpoint just says, “Here I have arrived at a foundation of all my beliefs. This position I will *hold*” (Wittgenstein 1969: sec. 246). “Giving grounds, […], justifying the evidence, comes to an end;—but the end is not certain propositions striking us immediately as true […] it is our *acting*, which lies at the bottom of the language-game” (Wittgenstein 1969: sec. 204).

The framework propositions of Christianity and its prayer are the “bedrock” that Wittgenstein describes or “what has to be accepted, the given” (Wittgenstein 1958: 226). The process of creating a foundation or justification ends when we are inclined to say: “This is simply what I do”. Wittgenstein notes: “At the foundation of well-founded belief lies belief that is not founded” (Wittgenstein 1969: sec. 253), and “the difficulty is to realize the groundlessness of our believing” (Wittgenstein 1969: sec. 166). “You must bear in mind that the language-game is so to say something unpredictable. I mean: it is not based on grounds. It is not reasonable (or unreasonable). It is there—like our life” (Wittgenstein 1969: sec. 559). The framework propositions of Christianity and its various forms of prayer cannot be, or do not have to be, founded or justified, and believers who devote themselves to prayer would say, “this is simply what we do”. Prayer is the linguistic expression of a believer’s commitment to God. As Wittgenstein states: “a language-game is only possible if one trusts something” (Wittgenstein 1969: sec. 509), a religious language-game, too, is possible only if a believer trusts or believes in God.

## Concluding Remarks

In the preceding paragraphs, we have presented insights of Schutz, Wittgenstein, Austin and Evans in order to approach the phenomenon of prayer from both a socio-phenomenological and a linguistic point of view. Following the linguistic strand, it has become evident that praying—understood as a performative or self-involving activity carried out in acts of praising, confessing, thanksgiving, or requesting to God—plays a basic role in the constitution of one’s religious life-world. In this context, we have interpreted prayer in Christianity as “a personal speaking, dialogue or communion with God” put into practice by following a specific set of rules. As Searle has pointed out in this regard, we may distinguish between two types of rules: “regulative rules” and “constitutive rules”. Though we tend to observe the former in everyday life, we should put an emphasis on the latter in our analysis, since they “create or define new^13^ forms of behavior” (see Searle 1980: 33–42). And in fact, Christian prayer, viewed as a groundless language-game, which follows a specific set of rules, is constitutive inasmuch as it (re)constitutes the correlated “reality” or “religious finite province of meaning” amidst the world of daily life.

In other words, the speech of prayer has its lasting tenor of meaning, and, hence, the question arises as to its relationship with other language-games in which Christians *nolens volens* participate in ordinary life. And in fact, the activity of prayer is carried out within the paramount reality of the everyday life-world, yet it results in the constitution of another, i.e., religious “finite province of meaning”. According to Okumura and others, who interpreted prayer as a “breaking off [of] all relations with daily life,” this reality is closed or cut off from other language-games. Though Bochenski and Sherry suggest that there is a complicated interplay between sacred or religious terms/discourse and profane or ordinary terms/discourse in question (see footnote 12), a linguistic account might insist on the “within-ness,” “totality,” or “wholeness” of prayer as a language-game in order to shed new light on understanding its logical structure. Against this backdrop, we could say that a person praying is playing an “in-game” which is autonomous or self-contained but has no means to relate itself to other language-games. It is in this context, however, that a recourse to Schutz and his theory of the symbol and its eminent role in the constitution of “multiple realities” may provide a more convincing description of this situation.

As we have already emphasized, Schutz insists, too, on the fact that “there is no possibility of referring one of these provinces to the other by introducing a formula of transformation” (Schutz 1945a: 232). Schutz, however, tackles the problem of communication that arises in face of this lack of a “formula of transformation” by implementing his theory of the symbol. Put in a nutshell, he interprets “symbol” as “an appresentational reference of a higher order” as follows:

A symbol can be defined […] as an appresentational reference of a higher order in which the appresenting member of the pair is an object, fact, or event within the reality of our daily life, whereas the other appresented member of the pair refers to an idea which transcends our experience of everyday life. (Schutz 1945b: 331, 343, 337-339)

The language employed in prayer is, without a doubt, as a whole “symbolic” in the Schutzian sense of the word. In the context of this paper, for instance, the word “God” used in a prayer represents an appresenting “object” used within the reality of our daily life and “something” appresented by the word is an “idea” which transcends the experiential framework of everyday life. Linguistic activities of praying, such as praising, confessing, thanksgiving, or requesting to God, interlink “appresenting” and “appresented” aspects. In other words, the scheme of “symbolic appresentation” points at the “indicating quality of the sign” (or the signifier), but must not be confused with the “scheme of reference,” which refers to *what* is indicated by the sign (the signified) (see Srubar 2014: 86). In light of this distinction, we may argue, following Srubar, “that Schutz was not only thinking of a theory of signs, but rather of a theory of the semiotic order of the life-world. For Schutz, the semiosis is thus the constituting force of the life-world, through which the different realities of the life-world as well as their constitutive levels are related to, and interwoven with, each other” (Srubar 2014: 86).

Given this, the basic point is not at all about finding a “formula of transformation”; it is not about translating one “reality” into another but rather about re-lating one such reality to itself *in the light* of another, i.e., to enable a different, self-reflective and critical way of relating to itself (and its assumed totality, rationality, truth, etc.). Put differently, prayer, which embodies and indeed epitomizes a sort of “religious epoché,” constitutes, on the one hand, an “immanent frame” of a closed language-game and, on the other hand, also allows one to “see through,” as Schutz put it, the immanent order of the paramount reality of the everyday life-world.

The way Schutz points out a problem of communication between a phenomenologist and a man of the “natural attitude” is revealing in this regard:

After having performed the phenomenological reduction, the phenomenologist finds himself confronted with the difficulty of communicating his knowledge to the “dogmatist” who remains within the natural attitude. Does not this presuppose a common ground between them? (Schutz 1945a: 256f.)

To our understanding, the phenomenologist’s standpoint is the key to the solution of our difficulty. He does not have to leave the “transcendental attitude” and return to the natural one. The difficulty disappears, as Schutz says, if “he places himself “*in*” the natural attitude as a transcendental situation that is seen through by him” (Schutz 1945a:257).

This argument is full of suggestiveness to combine Schutzian phenomenology and linguistic philosophy with a view to arrive at a deeper understanding of the topic at hand. This specifically Schutzian move helps to get a better and deeper grip on the *social* *embodiment* of prayer in terms of a performative symbolic act. And indeed, man does not pray to the “causa sui,”which is too abstract as an object of prayer, i.e., before which, as Heidegger famously put it, one could “neither fall to his knees in awe nor can he play music and dance” (Heidegger 1969: 72) Schutz starts, in a quite similar way, from the idea of an “embodied intentionality” or a person who accesses another reality in praying, which enables him/her to “see through” the world and the relevancies of daily life and let it appear in a different light, e.g., the light of grace, gift, or salvation. The predominant pragmatic infrastructure and constitution of the everyday world and its foundational motive, the so-called “fundamental anxiety,” (Schutz 1945a: 228), thus, is substituted by a different, anti-pragmatic logic, i.e., one which revolves around the ultimate “groundlessness” of this existence and our obligation and giftedness to take over responsibility for it.^14^

As we have attempted to demonstrate, phenomenology and linguistic philosophy can team up productively when being applied to the analysis of “prayer”. Prayer, consisting of a conscious aspect and a linguistic one, emerges in the world of daily life and constructs its “field” or “enclave” in the midst of this world. Put differently, it jumps out of the pre-determined horizons of the flow of consciousness into the living core of experience, thus opening our self to experiencing forms of transcendence that exceed the horizontal structure of experience towards “vertical modes of givenness” (Steinbock 2007).

Prayer, to finally underscore this once more, is not about the social construction of access to some different ontological reality: the intelligibility of the “vertical world” derives, as Merleau-Ponty puts it, from *this* world. We rather have to conceive it as a fundamental act in the meaningful constitution of a religious life-world, and it does so in promoting a specific attitude, a style of experiencing, i.e., by what we have termed a “religious epoché”. The (re)enactment of this “epoché” is facilitated by (but not dependent upon) the creation of an “enclave” in the everyday world (the church, the temple, the sacred site), where symbolic patterns of interaction and communication (“i.e., symbolic appresentation”) are socially derived, routinized, and, at least to some extent, institutionalized (ritualized). Thus viewed, Schutz’s account indeed offers a productive supplement to a linguistic approach. In this context, his theory of the symbol—and language in general—is of paramount importance: in conceiving the symbol as a sort of “meaning-clip” (Srubar 2007: 201–3) which allows us to relate “multiple realities,” the symbolism of prayer creates an “enclave” in everydayness that makes this symbolic world tick—giving rise to think always more, i.e., beyond the unanimity of a world governed by the “pragmatic motive”.

## Footnotes

1 All quotations from the Bible are based on the New Revised Standard Version.

2 As for the respective importance of collective/communal prayer and individual/solitary prayer, Chrétien provides the reader with a brief historical survey (Chrétien 2004: 33f.) and points out: “the superiority of collective prayer is emphasized by numerous religious traditions” (Chrétien 2004: 33) and “In Christianity […] all prayer is in essence communal, since every individual prays and can pray […] only as a member of the Mystic Body of Christ, and thus always within the Church” (Chrétien 2004: 34). In terms of our phenomenological perspective employed here, however, individual/solitary prayer is also important because, methodologically regarded, the analysis of collective/communal prayer presupposes access to the level of individual/solitary consciousness and the meaning-bestowing activities that occur in the stream of consciousness. The “superiority of collective prayer,” thus viewed, has to be understood as an *existential* superiority. In other words, even motionless or silent prayer in the remoteness of, e.g., ascetic seclusion is but a modality of its basic sociality—but one that derives its significance from its individual articulation.

3 As regards our overall topic, an interesting example for the “bridging of multiple realities” from within the specific enclave of “computer religious worlds” is given by Waltemathe (2014). A more general presentation of the concept with reference to *art* as an “enclave of meaning” is provided by McDuffie (1995).

4 All the quotations from Okumura’s work are based on Hoshikawa's provisional translation (K. H.).

5 The experience of the “wholly other” may, of course, be qualified in infinitely different ways, like, e.g., with regard to the ultimate futility of all our mundane achievements and our finitude, the non-identifiable call of the other person, the sublimity of nature that we are used to exploit, or the gift-character of all reality, etc. As for our context, it is important to note that this experience of transcendence is not a self-sufficient experience but is rather intrinsically related to a self-transcendence of the everyday relevancies that are imposed on us. In other words, only in responding to the call of the “wholly other” we learn to “see through” the relevancies we always already live in without reflecting upon them. (Prayer, as we argue, is one way to articulate this transcendence which we are.) In this context, Schutz’s concept of relevance would, without a doubt, deserve further analysis.

6 In this context, we might refer to Husserl's concept of “Einströmen,” (“influx,” “flowing into”) (see Husserl 1970: 210) which he coined in order to demonstrate how the experience lived through in the transcendental attitude is structurally retained after returning to the “natural attitude,” thus modifying and enriching it in an irrevocable way.

7 Chrétien, criticizing G. Van der Leeuw’s book *Religion in Essence and Manifestation* (Van der Leeuw 1964) which distinguishes prayer from adoration, stresses: “The link between desire [or request] and prayer is essential, and it can serve to unify the various definitions that have been given of the latter” (Chrétien 2004: 29).

8 Chrétien proposed a phenomenological discussion on the contrast between “vocal” prayer and “silent” prayer (Chrétien 2004: 31–33). In antiquity, “praying aloud, clearly and intelligibly, is the most normal and most common kind of prayer” (2004: 32) and “Vocal prayer puts an end to the disorder of the murmurous noise within […]. The voice is not [merely] instrumental for prayer” (2004: 33). According to his outlook, the voice of prayer is its essence. Though we generally agree with him, silent/murmurous prayer is still acceptable, since it is but a variation of vocal prayer.

9 One might insist that when one speaks to oneself in prayer that s/he believes in God, s/he has no intention at all to prove the existence of God ontologically but that the existence of God would be rendered objectively valid. In other words, when one enunciates that the holy God be praised, then God is rendered existent objectively for the person praying. Given that, in a situation when the person praying is not making a factual statement by expressing his/her belief in God, s/he is not at all claiming that his/her assertion is only subjectively valid to himself/herself. In responding to this objection, we propose to understand “God” as “an intentional object” that is constructed by language” (see sec. 2). In other words, we propose not to commit ourselves at this point to an ontological or theological argument concerning the existence of God; this is beyond our analysis: “God” might or might not exist objectively. Even if the believer pretends to render the existence of God “objectively valid,” it is not “objective” in the strict sense of the word, because atheists or non-Christians may not accept the objective existence of God. But in any case the existence of God is still construed as an “intentional object,” since even atheists or non-Christians imagine “God” as an “intentional object”. Though the logic of self-involvement sounds “subjective,” it is our frame of reference not on the level of object-language but on the level of meta-language.

10 Wittgenstein clearly distinguishes between the “sayable/speakable” world, which is the world of facts, and the “unsayable/unspeakable” world, which is the world of absolute value or religion. He personally tried to bridge these two worlds by praying and took a serious view of prayer throughout his life.

11 It is impossible to explicitly identify all of the rules that are followed by believers. As Searle notes, this is not necessary since: “It seems obvious to me that it is a rule, and that it is one which we follow without necessarily knowing (in the sense of being able to formulate) that we do. […] Sometimes in order to explain adequately a piece of human behavior we have to suppose that it was done in accordance with a rule, even though the agent himself may not be able to state the rule and may not even be conscious of the fact that he is acting in accordance with the rule” (Searle 1980: 42).

12 Our understanding of prayer as an activity in a “finite province of meaning,” which is cut off from daily language-games, might provoke some objections. It is a serious and important question whether or not the language of prayer can be broken off from the language-game of daily affairs. For example, Bochenski, a famous logician, contends that “RD [sc., religious discourse] is very closely connected with TD [sc., total discourse] of its user, that is, it cannot be disconnected and considered separately from the PD [sc., profane discourse] of the same subject. Therefore, the problem of the relations between the two discourses, the RD and the PD, is here particularly important” (Bochenski 1965: 58). Additionally, Sherry categorizes terms used in Christianity into four groups: (1) Specifically religious terms like ‘God,’ ‘Holy Trinity,’ and ‘Hell’ in Christianity. (2) Metaphysical terms, for example, ‘omniscient,’ ‘infinite,’ or ‘spirit’. (3) Analogical terms, that is, terms from ordinary language used outside of their normal contexts, such as ‘father,’ ‘cause,’ ‘make,’ ‘redeem,’ ‘real,’ and ‘truth’. (4) Ordinary terms, for example, ‘death,’ ‘peace,’ ‘crucify,’ and common words like ‘and,’ ‘but,’ ‘all,’ and ‘not’ (see Sherry 1977: 57). Terms belonging to categories (2), (3), and (4) are also employed in non-Christian contexts, and all of the terms within the four categories are intermingled. However, if we focus on the “system,” “totality,” and “wholeness” of a language-game, as Wittgenstein stresses, it *is* possible to consider words, phrases, or sentences as forming their own language-game.

13 “New” in our context does not necessarily mean “not existing before”. It means that every time a believer devotes himself/herself to prayer, s/he constructs a finite province of meaning with its own cognitive style in the midst of daily life, even if the content of the prayer is the same as a previous one. It happens, of course, that a literally “new” prayer comes into existence once it is expressed.

14 On “anti-pragmatic motives” in Schutz see Barber’s paper in this issue (Barber 2015).
